# Trends in 10-Year Predicted Risk of Cardiovascular Disease Associated With Food Insecurity, 2007–2016

**DOI:** 10.3389/fcvm.2022.851984

**Published:** 2022-05-24

**Authors:** Parija Sharedalal, Neal Shah, Jayakumar Sreenivasan, Liana Michaud, Anmol Sharedalal, Risheek Kaul, Julio A. Panza, Wilbert S. Aronow, Howard A. Cooper

**Affiliations:** ^1^Department of Cardiology, Westchester Medical Center and New York Medical College, Valhalla, NY, United States; ^2^Department of Medicine, Westchester Medical Center and New York Medical College, Valhalla, NY, United States; ^3^School of Medicine, St. George’s University, St. George’s, Grenada

**Keywords:** prevention, food insecuirty, nutrition, cardiovascular health, cardiovascular prevention

## Abstract

**Introduction:**

Consumption of a healthy diet improves cardiovascular (CV) risk factors and reduces the development of cardiovascular disease (CVD). Food insecure (FIS) adults often consume an unhealthy diet, which can promote obesity, type 2 diabetes mellitus (T2DM), hypertension (HTN), and hyperlipidemia (HLD). The Supplemental Nutrition Assistance Program (SNAP) is designed to combat food insecurity by increasing access to healthy foods. However, there is a paucity of data on the association of SNAP participation among FIS adults and these CVD risk factors.

**Methods:**

The National Health and Nutrition Examination Survey (NHANES) is a publicly available, ongoing survey administered by the Centers for Disease Control and Prevention and the National Center for Health Statistics. We analyzed five survey cycles (2007–2016) of adult participants who responded to the CVD risk profile questionnaire data. We estimated the burden of select CVD risk factors among the FIS population and the association with participation in SNAP.

**Results:**

Among 10,449 adult participants of the survey, 3,485 (33.3%) identified themselves as FIS. Food insecurity was more common among those who were younger, female, Hispanic, and Black. Among the FIS, SNAP recipients, when compared to non-SNAP recipients, had a lower prevalence of HLD (36.3 vs. 40.1% *p* = 0.02), whereas rates of T2DM, HTN, and obesity were similar. Over the 10-year survey period, FIS SNAP recipients demonstrated a reduction in the prevalence of HTN (*p* < 0.001) and HLD (*p* < 0.001) which was not evident among those not receiving SNAP. However, obesity decreased only among those not receiving SNAP. The prevalence of T2DM did not change over the study period in either group.

**Conclusion:**

Over a 10-year period, FIS adults who received SNAP demonstrated a reduction in the prevalence of HTN and HLD, which was not seen among those not receiving SNAP. However, the prevalence of obesity and T2DM did not decline among SNAP recipients, suggesting that additional approaches are required to impact these important CVD risk factors.

## Introduction

Cardiovascular disease (CVD) is a major global health challenge, and was responsible for 23% of total deaths in the United States (US) in 2017 (1). Consumption of a healthy and well-balanced diet is associated with better cardiovascular (CV) outcomes and has been demonstrated to improve CVD risk factors through decrease in body weight and various cardiometabolic pathways (2). Current guidelines emphasize a diet composed of fruits, vegetables, legumes, fish, nuts, whole grains, and fibers along with a reduction in sodium, processed meats, sugar-sweetened beverages (SSB), excess calories and refined foods (2, 3).

The US Department of Agriculture (USDA) defines food insecurity as economic and social conditions associated with limited and uncertain access to food (4). In 2018, 37.2 million Americans lived in food insecure (FIS) households (4). Inadequate financial resources, the high cost of food, and competing priorities often cause adults with food insecurity to consume an unhealthy and low quality diet (5–7). FIS adults often forgo purchasing specialized diets for medical conditions such as hypertension (HTN) or type 2 diabetes mellitus (T2DM) in favor of purchasing low-cost foods that are often energy dense but nutrient poor (8). As a result, food insecurity can lead to worsening of CVD risk factors including T2DM, HTN, hyperlipidemia (HLD), and obesity defined as BMI ≥ 30 kg/m^2^ (9), thus contributing to poor CV health.

The Supplemental Nutrition Assistance Program (SNAP, formerly called Food Stamps Program) is the largest federal food assistance program available for low-income households, and is designed to directly combat food insecurity (10). SNAP eligibility is based on household income, specifically targeting low income Americans living under the poverty level (11). Access to SNAP can potentially address high rates of CVD risk factors in FIS adults by increasing access to healthy foods. While few studies have shown a link between CVD risk factors and food insecurity (12, 13), there is a paucity of data on the association of SNAP participation amongst FIS adults and CVD risk factors. Understanding this relationship is important to determine whether any future expansions, additions or modifications to the SNAP program are needed to better serve the FIS population in the United States (US). We designed this study to investigate the prevalence of CV risk factors among the FIS population, and analyze the association between participation in SNAP and the change in CVD risk factors over a 10-year study period.

## Materials and Methods

### Data Source

The National Health and Nutrition Examination Survey (NHANES) is an ongoing cross-sectional survey administered by the Centers for Disease Control and Prevention (CDC) and the National Center for Health Statistics (NCHS). It is designed to describe the health and nutritional status of the non-institutionalized population of the United States (14). The NHANES database is publicly available for researchers, and the data are released in 2-year cycles. All NHANES participants complete an English or Spanish language questionnaire during a home interview. Participants then undergo a standardized physical examination (including height, weight).

### Food Security

The food security module within NHANES is a questionnaire developed by the USDA to measure adult food security over the 12 months preceding the survey. Adult food security is categorized by NHANES as: full food security, marginal food security, low food security, and very low food security. In this study, we considered adults in the full food security category to be food secure (FS) and those categorized as having marginal, low and very low food security to be FIS.

### Study Population

We analyzed 10-year data from five survey cycles completed by adult participants ≥ 18 years of age, from months of January to December (2007–2008, 2009–2010, 2011–2012, 2013–2014, and 2014–2016). A total of 10,449 adults who participated in the NHANES surveys from 2007 to 2016 were included in the full analytic sample. From this cohort, 6,964 adults were identified as FS (66.6%), and 3,485 adults were identified as FIS (33.3%). The FIS adult participants were further categorized into those receiving SNAP benefits (63.3%) and those not receiving SNAP benefits (36.6%).

The presence or absence of each CVD risk factor (T2DM, HTN, HLD, obesity) was determined based on the response to the survey question: “Have you ever been diagnosed by a physician with this risk factor?” Respondents who were classified as SNAP participants, had accepted SNAP benefits in the 12 months preceding the survey.

### Statistical Analysis

All analyses were performed using SPSS, version 26 (IBM, Chicago). Stratified weighted data were utilized to obtain nationwide estimates. Continuous variables were reported as mean ± standard deviation and compared using the Student *t*-test, and the categorical variables were reported as proportions and compared using the Chi-square test. We used multiple logistic regression analysis to determine temporal trends with survey year as the independent variable and the CV risk factor of interest as the dependent variable. The yearly prevalence of various CV risk factors among the study cohorts was reported along with P-*trend.*

## Results

**FIGURE 1:**
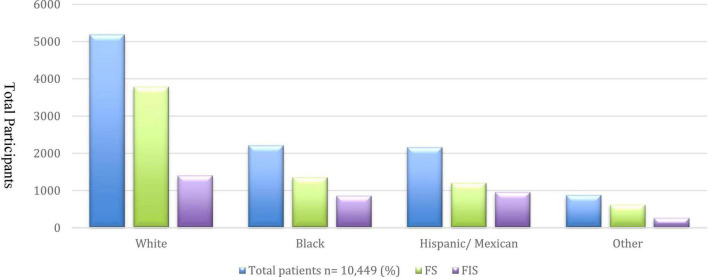
Food insecurity in the total population categorized by ethnicity/race.

A total of 10,449 adults (mean age 53.16 ± 16.9, 41.6% females) who participated in the NHANES surveys from 2007 to 2016 were included in the full analytic sample. From this cohort, 6,964 adults were identified as FS (66.6%), and 3,485 adults were identified as FIS (33.3%). The prevalence of CVD risk factors in the total population, and according to food security status is shown in Table 1. CVD risk factors were highly prevalent among all participants. FIS adults were younger than those who were FS. The proportion of females (Table 1) and self-identified Blacks and Hispanics was higher among FIS participants than FS participants of the survey (Figure 1). The prevalence of HLD and obesity were significantly higher among those with FIS, whereas rates of HTN and T2DM were similar in the two groups.

**TABLE 1:** Baseline characteristics of the total adult participants of the survey, 2007–2016^a,b^.

Population characteristics	Total patients *n* = 10, 449 (%)	FS, *n* = 6, 964 (%)	FIS, *n* = 3, 485 (%)	*P*-value
Age	53.16 ± 16.9	55.75 ± 16.8	48.0 ± 16.1	< 0.00
HTN	4581 (43.8)	3085 (44.3)	1496 (42.9)	0.183
HLD	4162 (39.8)	3048 (43.8)	1114 (31.9)	0
T2DM	1638 (15.7)	1060 (15.2)	578 (16.5)	0.071
Obesity^c^	4057 (38.8)	2549 (36.6)	1508 (43.3)	< 0.00
Female	4354 (41.6)	2776 (39.9)	1578 (45.3)	< 0.01
**Race**				
White	5192 (49.7)	3787 (54.3)	1405 (40.3)	< 0.00
Black	2213 (21.2)	1353 (19.4)	860 (24.7)	< 0.00
Hispanic/Mexican	2165 (20.7)	1206 (17.3)	959 (27.5)	< 0.00
Other	879 (8.4)	618 (8.9)	261 (7.4)	< 0.00

*HTN, hypertension; HLD, hyperlipedemia; T2DM, type 2 diabetes mellitus; SNAP, Supplement Nutrition Assistance Program; FS, Food Secure; FIS, Food Insecure. All relationships are statistically significant for p < 0.001. Categorical variables are reported as proportions and compared using the Chi-square test. Continuous variables such as age is reported as mean ± standard deviation and compared using the Student t-test.*

*^a^Data from the National Health and Nutrition Examination Survey.*

*^b^Data were weighted to be nationally representative.*

*^c^Obesity defined as BMI (calculated as weight in kilograms divided by height in meters squared) ≥ 30 kg/m.*

**FIGURE 2:**
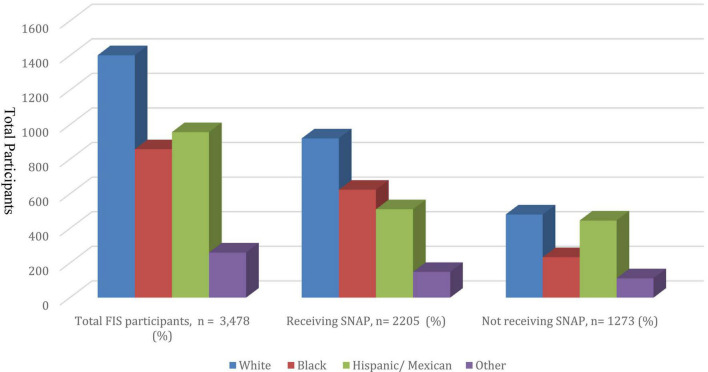
SNAP participation in FIS population categorized by ethnicity/race.

Characteristics of the FIS participants according to receipt of SNAP benefits are described in Table 2. Those receiving SNAP comprised 63.4% of the FIS population. SNAP participants were younger and more likely to be female and non-white. Among the FIS, higher proportion of self-identified whites (41.8%), participated in SNAP compared to self-identified Hispanics/Mexicans (23.2%) (Figure 2). As described in Table 2, FIS participants who received SNAP had a lower prevalence of HLD compared to those who did not receive SNAP (36.3 vs. 40.1%, *p* = 0.02). There was no significant difference in the prevalence of T2DM, HTN, or obesity between FIS participants receiving SNAP and FIS participants not receiving SNAP.

**TABLE 2:** Baseline characteristics of the adult food insecure participants of the survey, 2007–2016^a–b^.

Population characteristics	Total FIS participants, *n* = 3, 478 (%)	Receiving SNAP, *n* = 2205 (%)	Not receiving SNAP, *n* = 1273 (%)	*P*-value
Age	47.99 ± 16.1	46.55 ± 15.67	50.49 ± 16.5	< 0.00
HTN	1492 (42.9)	956 (43.3)	536 (42.1)	0.473
HLD	1311 (37.7)	800 (36.3)	511 (40.1)	0.024
T2DM	576 (16.6)	346 (15.7)	230 (18.0)	0.069
Obesity^c^	1503 (43.2)	970 (44)	533 (41.9)	0.224
Female	1574 (45.2)	1079 (48.9)	495 (38.9)	< 0.00
**Race**				
White	1402 (40.3)	921 (41.8)	481 (37.8)	< 0.00
Black	858 (24.7)	624 (28.3)	234 (18.4)	< 0.00
Hispanic/Mexican	957 (27.5)	511 (23.2)	446 (35.0)	< 0.00
Other	261 (7.5)	149 (6.7)	112 (8.8)	< 0.00

*HTN, hypertension; HLD, hyperlipedemia; T2DM, type 2 diabetes mellitus; SNAP, Supplement Nutrition Assistance Program; FIS, Food Insecure. Continuous variables such as age is reported as mean ± standard deviation and compared using the Student t-test; categorical variables are reported as proportions and compared using the Chi-square test. All relationships are statistically significant for p < 0.001.*

*^a^Data from the National Health and Nutrition Examination Survey.*

*^b^Data were weighted to be nationally representative.*

*^c^Obesity defined as BMI (calculated as weight in kilograms divided by height in meters squared) ≥ 30 kg/m.*

Among all NHANES participants, the prevalence of HTN and HLD decreased over the 10-year study period (Table 3). Rates of T2DM and obesity remained unchanged over the study period in the total population (Table 3). When analyzed according to food security status, similar trends were seen in both FS and FIS groups (Table 4). The prevalence of HTN and HLD showed statistically significant decrease over the study period in both FS and FIS groups (Table 4).

**TABLE 3:** Trends of CVD risk factors of the total adult participants of the survey, 2007–2016^a–b^.

	2007–2008 *n* = 1839 (%)	2009–2010 *n* = 1850 (%)	2011–2012 *n* = 2161 (%)	2013–2014 *n* = 2404 (%)	2015–2016 *n* = 2195 (%)	*p*-value
HTN	884 (48.1)	920 (49.7)	880 (40.7)	988 (41.4)	909 (41.4)	< 0.00
T2DM	309 (16.8)	316 (17.1)	316 (14.6)	332 (13.8)	365 (16.6)	0.173
HLD	909 (49.4)	890 (48.1)	821 (38)	904 (37.6)	838 (38.2)	< 0.00
Obesity^c^	718 (39)	786 (42.5)	764 (35.4)	896 (37.3)	893 (40.7)	0.649

*HTN, hypertension; HLD, hyperlipedemia; T2DM, type 2 diabetes mellitus; BMI, Body Mass Index; CVD, cardiovascular disease. Multiple logistic regression analysis was used to determine temporal trends with survey year as the independent variable and the CVD risk factor of interest as the dependent variable. All relationships are statistically significant for p < 0.001.*

*^a^Data from the National Health and Nutrition Examination Survey.*

*^b^Data were weighted to be nationally representative.*

*^c^Obesity defined as BMI (calculated as weight in kilograms divided by height in meters squared) ≥ 30 kg/m.*

**TABLE 4:** Trends in CVD risk factors of adult food insecure participants of the survey, 2007–2016^a,b^.

		2007–2008 *n* = 1418 (%)	2009–2010 *n* = 1320 (%)	2011–2012 *n* = 1392 (%)	2013–2014 *n* = 1572 (%)	2015–2016 *n* = 1262 (%)	*p*-value
HTN	FS	678, (47.8)	649 (49.2)	576 (41.4)	664 (42.2)	518 (41)	< 0.001
	FIS	206 (48.9)	271 (51.5)	304 (39.5)	324 (38.9)	391 (41.9)	< 0.001
T2DM	FS	233 (16.4)	221 (16.7)	194 (13.9)	222 (14.1)	190 (15.0)	0.07
	FIS	76 (18.0)	95 (17.9)	122 (15.9)	110 (13.2)	175 (18.7)	0.914
HLD	FS	701 (49.4)	630 (47.7)	559 (40.1)	646 (41.1)	512 (40.5)	< 0.00
	FIS	208 (49.4)	260 (49.0)	262 (34.0)	258 (31.0)	326 (34.9)	< 0.00
Obesity^c^	FS	527 (37.1)	530 (40.1)	443 (31.8)	557 (35.4)	492 (38.9)	0.708
	FIS	191 (45.4)	256 (48.3)	321 (41.7)	339 (40.7)	401 (42.9)	0.071

*HTN, hypertension; HLD, hyperlipedemia; T2DM, type 2 diabetes mellitus; SNAP, Supplement Nutrition Assistance Program; FS, Food Secure; FIS, Food Insecure; CVD, cardiovascular disease. Multiple logistic regression analysis is used to determine temporal trends with survey year as the independent variable and the CV risk factor of interest as the dependent variable. All relationships are statistically significant for p < 0.001.*

*^a^Data from the National Health and Nutrition Examination Survey.*

*^b^Data were weighted to be nationally representative.*

*^c^Obesity defined as BMI (calculated as weight in kilograms divided by height in meters squared) ≥ 30 kg/m.*

When FIS group was further analyzed according to participation in SNAP benefits, FIS SNAP participants demonstrated a significant reduction in the prevalence of HTN (*p* < 0.001) and HLD (*p* < 0.001) over the study period (Table 5). No significant change was noted in the prevalence of T2DM or obesity over 10 years (Table 5). Among the non-SNAP participants, HLD decreased significantly (*p* = 0.007) but HTN did not (Table 5). Remarkably, obesity rates decreased significantly only in the group not receiving SNAP (Table 5).

**TABLE 5:** Trends in CVD risk factors of adult food insecure participants of the survey, categorized by SNAP participation, 2007–2016 ^a–b^.

	Column 1	2007-2008 *n* = 219 (%)	2009–2010 *n* = 296 (%)	2011–2012 *n* = 461 (%)	2013–2014 *n* = 558 (%)	2015–2016 *n* = 671 (%)	*p*-value
HTN	SNAP	113 (51.6)	157 (53.0)	187 (40.6)	218 (39.0)	281 (48.9)	< 0.00
	NO SNAP	93 (46)	114 (48.7)	113 (37.4)	106 (38.8)	110 (41.9)	0.087
T2DM	SNAP	34 (15.5)	56 (18.9)	73 (15.8)	75 (13.4)	108 (16.0)	0.476
	NO SNAP	42 (20.8)	39 (16.7)	47 (15.6)	35 (12.8)	67 (25.6)	0.354
HLD	SNAP	108 (49.3)	154 (52.0)	158 (34.3)	157 (28.1)	223 (33.2)	< 0.00
	NO SNAP	100 (49.5)	106 (45.3)	102 (33.8)	100 (36.6)	103 (39.3)	0.007
Obesity^c^	SNAP	97 (44.2)	143 (48.3)	190 (41.2)	230 (41.2)	310 (46.2)	0.99
	NO SNAP	94 (46.5)	113 (48.3)	126 (41.7)	109 (39.9)	91 (34.7)	0.001

*HTN, hypertension; HLD, hyperlipedemia; T2DM, type 2 diabetes mellitus; SNAP, Supplement Nutrition Assistance Program; CVD, cardiovascular disease. Multiple logistic regression analysis is used to determine temporal trends with survey year as the independent variable and the CV risk factor of interest as the dependent variable. All relationships are statistically significant for p < 0.001.*

*^a^Data from the National Health and Nutrition Examination Survey.*

*^b^Data were weighted to be nationally representative.*

*^c^Obesity defined as BMI (calculated as weight in kilograms divided by height in meters squared) ≥ 30 kg/m.*

## Discussion

Our study provides important findings on the prevalence of CVD risk factors among FIS adults in the United States and the association with participation in SNAP.

The principal determinant of obesity and other cardiometabolic diseases is lifestyle, of which food habit is a main component (15). In our study, we found that the prevalence of obesity among FIS adults was higher compared to FS adults. Additionally, FIS SNAP participants did not show an improvement in the prevalence of obesity over 10 years. An interesting cross-sectional analysis by Myers et al. (16) from years 1999 to 2016 shows that prevalence of food insecurity has increased over the study period. Additionally, the study found that obese individuals had higher reports of food insecurity. Our study did not show statistically significant difference in prevalence of T2DM between FS and FIS groups. Similar prior studies (17, 18) however have shown association of food insecurity with poor glycemic and overall metabolic control in adults. Further studies are needed that examine association between food insecurity and CVD risk factors. Specific population subgroup analysis based on ethnicity, sex, and other social variables including economic, education level, geographic location, and marital status, can help identify factors which may reduce prevalence of both food insecurity and obesity in the long term.

Supplemental Nutrition Assistance Program is a federal program which offers financial assistance to eligible people, and is designed to strengthen food and nutritional security. Although previous reports (19) demonstrated that SNAP alleviates food insecurity and improves dietary quality, SNAP participants may in fact have lower quality diet than non-SNAP participants (20). While previous studies have shown that SNAP participation is effective in increasing intake of caloric and nutritious foods, SNAP participants overall do not meet the key recommended nutrition guidelines in daily food habits (20). Greater emphasis is needed to improve the diet quality, and subsequently the CV health of SNAP participants. Further, health care providers should be trained to screen for food security and have knowledge on how to address this topic with their patients.

There was a significant downward trend in the prevalence of HTN and HLD among the FIS SNAP participants over 10 years. Unhealthy diet is one of the top contributors for worsening CVD and associated risk factors (21). SNAP improves access of nutritionally adequate foods, and therefore has the capacity to influence household wellbeing beyond just nutrition and hunger, by directly impacting health outcomes of participants (2, 10). Despite this favorable trend in HTN and HLD in our study, prior studies have shown disparities in the diet quality between SNAP participants and the high income population (22). United States fruit and vegetable consumption in the low-income population remains below recommended levels (23). SNAP-Ed program is a USDA program which promotes healthier eating choices with a limited budget through education and awareness (24). The education programs, national campaigns from groups like American Heart Association (AHA) (25), and increase in public awareness on nutrition have shown an improvement in diet components of SNAP participants, including increase in whole grain, fruits, vegetables and decrease in SSB consumption (26). SNAP benefits are critical for many FIS low income families. Along with improving public education on nutrition, urgent reform and policy changes at state and local level that promote and support a healthier diet are needed. Mozaffarian and colleagues (27) who studied a model evaluating one such possible policy disincentivizing unhealthy food purchases, and incentivizing healthy food purchases including fruits and vegetables, noted a significant decrease in CV deaths and substantial cost benefits. More public health research assisting SNAP and other similar federal programs improving dietary quality of the participants is needed.

Our study has several limitations. First, as with all observational studies, our study cannot prove direct causality. Second, our data also does not allow for long-term assessment in SNAP participation and cardiovascular outcomes. Food security, depending on the adequacy of food, can wax and wane. As such, an individual’s level of food security, and therefore SNAP participation may change over time. The level of food security over time is not measured in our data, which focuses on the previous 12-months. Third, our analysis is based on the self-reported presence or absence of CVD risk factors, rather than actual measurement of these risk factors. Therefore, some misclassification is possible. Fourth, we were unable to exclude participants with established CVD, as the survey does not contain this information. Finally, NHANES only assesses responders’ food insecurity over the last 12 months before survey participation, and thus we were not able to assess long-term food security levels amongst survey participants.

## Conclusion

Ready access to guideline-recommended nutrient intake remains a challenge in the United States. Our study shows the favorable trends in the prevalence of HTN and HLD among FIS SNAP participants over a 10 year period. Our study also highlights the persistence of high levels of T2DM and obesity in the adult FIS population. These findings support the need for continuing efforts toward promoting a sustainable, healthy diet among the low-income population and the need to further reduce the health disparities caused by food insecurity.

## Data Availability Statement

Publicly available datasets were analyzed in this study. This data can be found here: NHANES, https://www.cdc.gov/nchs/nhanes/index.htm.

## Author Contributions

PS designed and directed this study and wrote the manuscript. HC, JP, and WA provided conceptual and technical guidance. NS and JS provided guidance with statistical analysis for this study. RK, LM, and AS provided support with literature review and editing and background researching the topic of food insecurity. All authors contributed to the article and approved the submitted version.

## Conflict of Interest

The authors declare that the research was conducted in the absence of any commercial or financial relationships that could be construed as a potential conflict of interest.

## Publisher’s Note

All claims expressed in this article are solely those of the authors and do not necessarily represent those of their affiliated organizations, or those of the publisher, the editors and the reviewers. Any product that may be evaluated in this article, or claim that may be made by its manufacturer, is not guaranteed or endorsed by the publisher.
